# Supplementary material for: Structure and Glass Transition Temperature of Amorphous Dispersions of Model Pharmaceuticals with Nucleobases from Molecular Dynamics
Source: Pharmaceutics. 2021 Aug 13;13(8):1253. doi: 10.3390/pharmaceutics13081253 (PMC8400648; doi:10.3390/pharmaceutics13081253)
Supplement: Supplementary file 1 [file pharmaceutics-13-01253-s001.zip › pharmaceutics-1342792-supplementary.pdf]

pharmaceutics

MDPI

Supplementary Materials: Structure and Glass Transition Temperature of Amorphous Dispersions of Model Pharmaceuticals with Nucleobases from Molecular Dynamics

Ctirad Červinka * and Michal Fulem

Table S1. Non-bonded force field parameters for carbamazepin.

Atom type	q_i / e	σ / Å	ε / kcal·mol^{-1}
CZ_1	-0.1469	3.800	0.115
HZC_1	0.0845	2.420	0.030
CZ_2	0.0993	3.550	0.070
CZ_3	0.2228	3.550	0.070
CZ_4	-0.1901	3.550	0.070
HZC_4	0.1164	2.420	0.030
CZ_5	-0.2418	3.550	0.070
HZC_5	0.1522	2.420	0.030
CZ_6	-0.0624	3.550	0.070
HZC_6	0.0843	2.420	0.030
CZ_7	-0.0874	3.550	0.070
HZC_7	0.0831	2.420	0.030
NZ_1	-0.2240	3.250	0.170
CZN	0.7340	3.750	0.105
OZ	-0.5974	2.960	0.210
NZ_2	-0.7624	3.250	0.170
HZ_2	0.3109	0.000	0.000

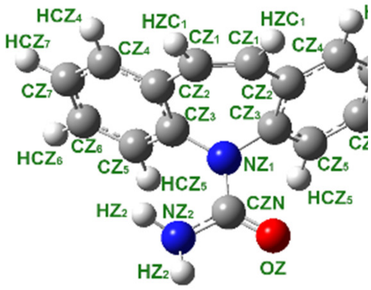

Table S2. Non-bonded force field parameters for ibuprofen.

Atom type	q_i / e	σ / Å	ε / kcal·mol^{-1}
CBA	-0.1150	3.550	0.070
HBA	0.1150	2.420	0.030
CB_1	0.0550	3.500	0.080
HBC	0.0600	2.500	0.080
CB_2	-0.0050	3.500	0.080
CB_3	-0.1800	3.500	0.080
CB_4	-0.0600	3.500	0.080
CB_5	-0.1800	3.500	0.080
CBO	0.5200	3.750	0.105
OB	-0.4400	2.960	0.210
OBH	-0.5300	2.960	0.210
HB	0.4500	0.000	0.000

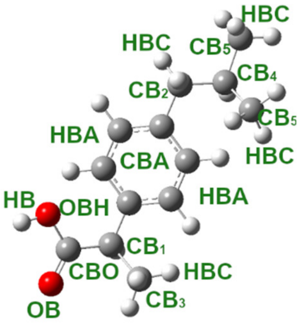

Table S3. Non-bonded force field parameters for indomethacin.

Atom type	q_i / e	σ / Å	ε / kcal·mol^{-1}
ND	-0.2105	3.250	0.170
CD_1	0.1333	3.550	0.070
CD_2	-0.1811	3.550	0.070
CD_3	0.0755	3.550	0.070
CD_4	-0.3321	3.550	0.070
CD_5	0.3645	3.550	0.070
CD_6	-0.3412	3.550	0.070
CD_7	-0.1605	3.550	0.070
CD_8	0.0232	3.550	0.070

CD$_9$	0.7459	3.750	0.105
CD$_{10}$	-0.2449	3.550	0.070
CD$_{11}$	-0.0774	3.550	0.070
CD$_{12}$	-0.0319	3.550	0.070
CD$_{13}$	-0.0081	3.550	0.070
CD$_{14}$	-0.3241	3.500	0.660
CD$_{15}$	-0.0272	3.500	0.660
CD$_{16}$	0.7025	3.750	0.105
CD$_{17}$	0.0973	3.800	0.170
ODE	-0.3810	3.000	0.170
OD$_1$	-0.5422	2.960	0.210
ODH	-0.6002	2.960	0.210
HD	0.4666	0.000	0.000
OD$_2$	-0.6049	2.960	0.210
ClD	-0.0987	3.400	0.300
HDC$_4$	0.1821	2.420	0.030
HDC$_6$	0.1837	2.420	0.030
HDC$_7$	0.1816	2.420	0.030
HDC$_{11}$	0.1223	2.420	0.030
HDC$_{12}$	0.1184	2.420	0.030
HDC$_{14}$	0.1242	2.500	0.030
HDC$_{15}$	0.0604	2.500	0.030
HDC$_{17}$	0.0481	2.500	0.030

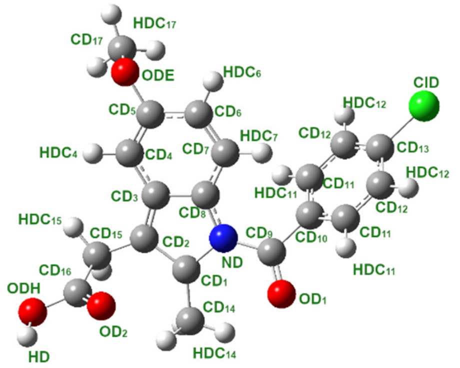

Table S4. Non-bonded force field parameters for naproxen.

Atom type	q_i / e	σ / Å	ε / kcal·mol^{-1}
CN1	-0.2292	3.550	0.070
CN2	-0.1421	3.550	0.070
CN3	0.1547	3.550	0.070
CN4	-0.3135	3.550	0.070
CN5	0.1471	3.550	0.070
CN6	-0.4278	3.550	0.070
CN7	0.4590	3.550	0.070
CN8	0.0840	3.500	0.080
CN9	-0.1316	3.500	0.066
CNE	0.1884	3.800	0.170
CNO	0.6794	3.750	0.105
ONE	-0.3923	3.070	0.170
ON	-0.5617	2.960	0.210
ONH	-0.6402	3.000	0.170
HN	0.4225	0.000	0.000
HNC1	0.1293	2.420	0.030
HNC2	0.1037	2.420	0.030
HNC4	0.1438	2.420	0.030
HNC6	0.1438	2.420	0.030
HNC8	0.0388	2.500	0.030
HNC9	0.0306	2.500	0.030
HNE	0.0119	2.500	0.030

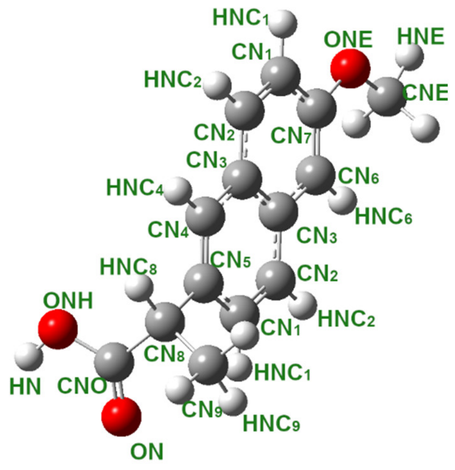

Table S5. Non-bonded force field parameters for adenine.

Atom type	q_i / e	σ / Å	ε / kcal·mol^{-1}
NA6$_1$	-0.7206	3.250	0.170
CA6$_1$	0.5255	3.500	0.080
NA6$_2$	-0.7227	3.250	0.170
CAJ$_1$	0.6052	3.500	0.080
CAJ$_2$	-0.0197	3.500	0.080
CA6$_2$	0.6940	3.500	0.080
NA5$_1$	-0.5550	3.250	0.170

CA5$_1$	0.3357	3.500	0.080
NA5$_2$	-0.5215	3.250	0.170
HA6$_1$	0.0360	2.500	0.050
NAH	-0.8357	3.250	0.170
HA2$_1$	0.3808	0.000	0.000
HA2$_2$	0.3655	0.000	0.000
HA5$_1$	0.0736	2.500	0.050
HA1	0.3589	0.000	0.000

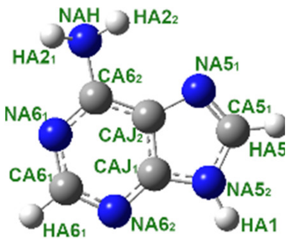

Table S6. Non-bonded force field parameters for cytosine.

Atom type	q_i / e	σ / Å	ε / kcal·mol^{-1}
NCH$_1$	-0.6154	3.250	0.170
CCO	0.9721	3.750	0.105
NCC	-0.8054	3.250	0.170
CCN	0.9886	3.500	0.080
CCC$_1$	-0.6107	3.500	0.080
CCC$_2$	0.2545	3.500	0.080
OC	-0.6338	2.960	0.210
NCH$_2$	-1.0307	3.250	0.170
HC1	0.3432	0.000	0.000
HC2$_1$	0.4298	0.000	0.000
HC2$_2$	0.4207	0.000	0.000
HCC$_1$	0.1857	2.500	0.050
HCC$_2$	0.1014	2.500	0.050

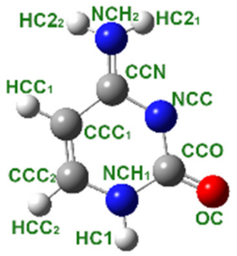

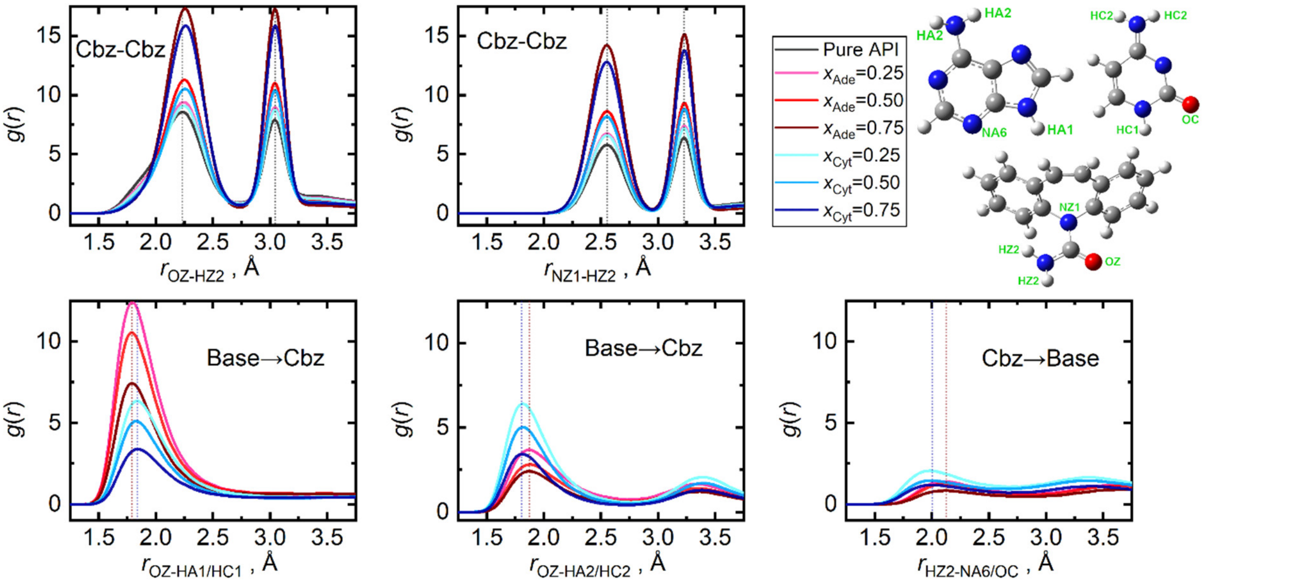

Figure S1. Radial distribution functions $g(r)$ at 410 K illustrating the structure of hydrogen bonding in pure amorphous carbamazepine (upper row) and in its mixtures with adenine or cytosine at various composition. Functions are displayed only for the most important contact sites in the given molecules.

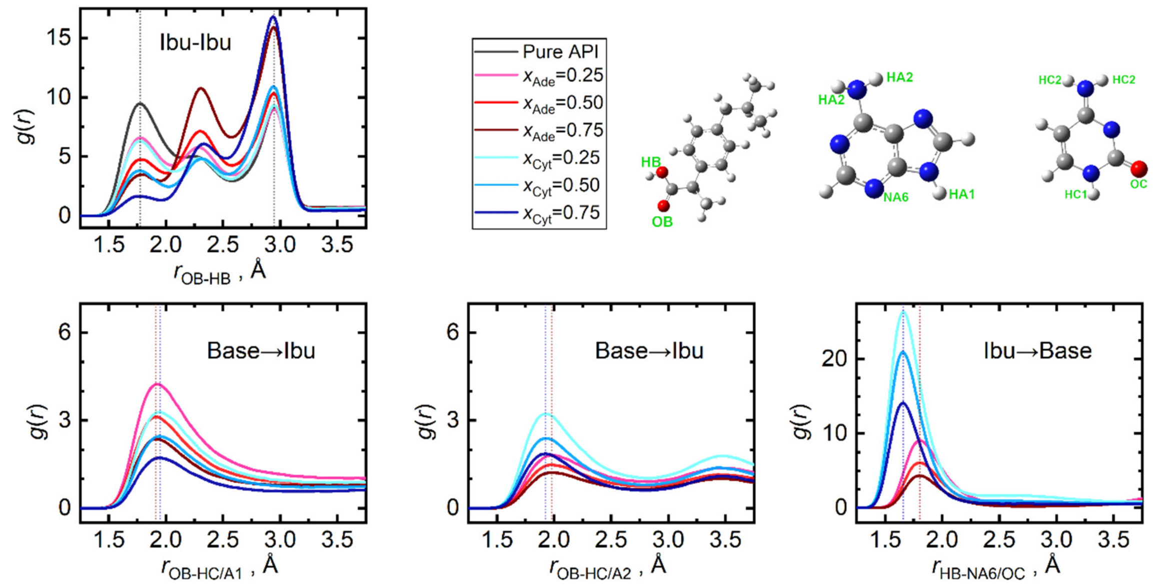

Figure S2. Radial distribution functions $g(r)$ at 410 K illustrating the structure of hydrogen bonding in pure amorphous RS-ibuprofen (upper row) and in its mixtures with adenine or cytosine at various composition. Functions are displayed only for the most important contact sites in the given molecules.

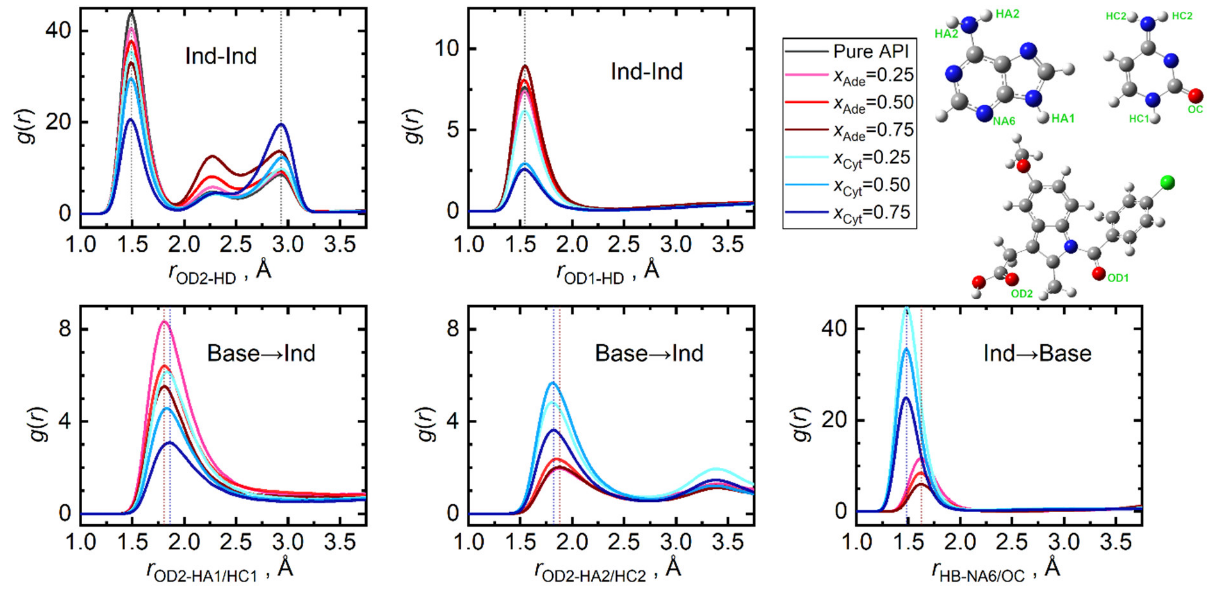

Figure S3. Radial distribution functions $g(r)$ at 410 K illustrating the structure of hydrogen bonding in pure amorphous indomethacin (upper row) and in its mixtures with adenine or cytosine at various composition. Functions are displayed only for the most important contact sites in the given molecules.

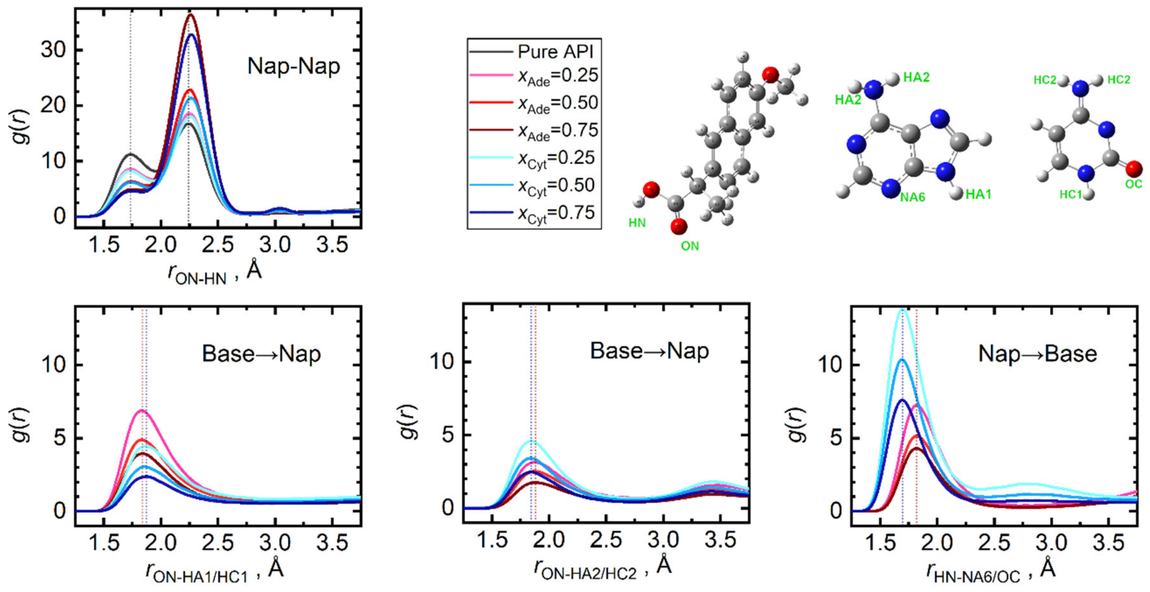

Figure S4. Radial distribution functions $g(r)$ at 410 K illustrating the structure of hydrogen bonding in pure amorphous naproxen (upper row) and in its mixtures with adenine or cytosine at various composition. Functions are displayed only for the most important contact sites in the given molecules.

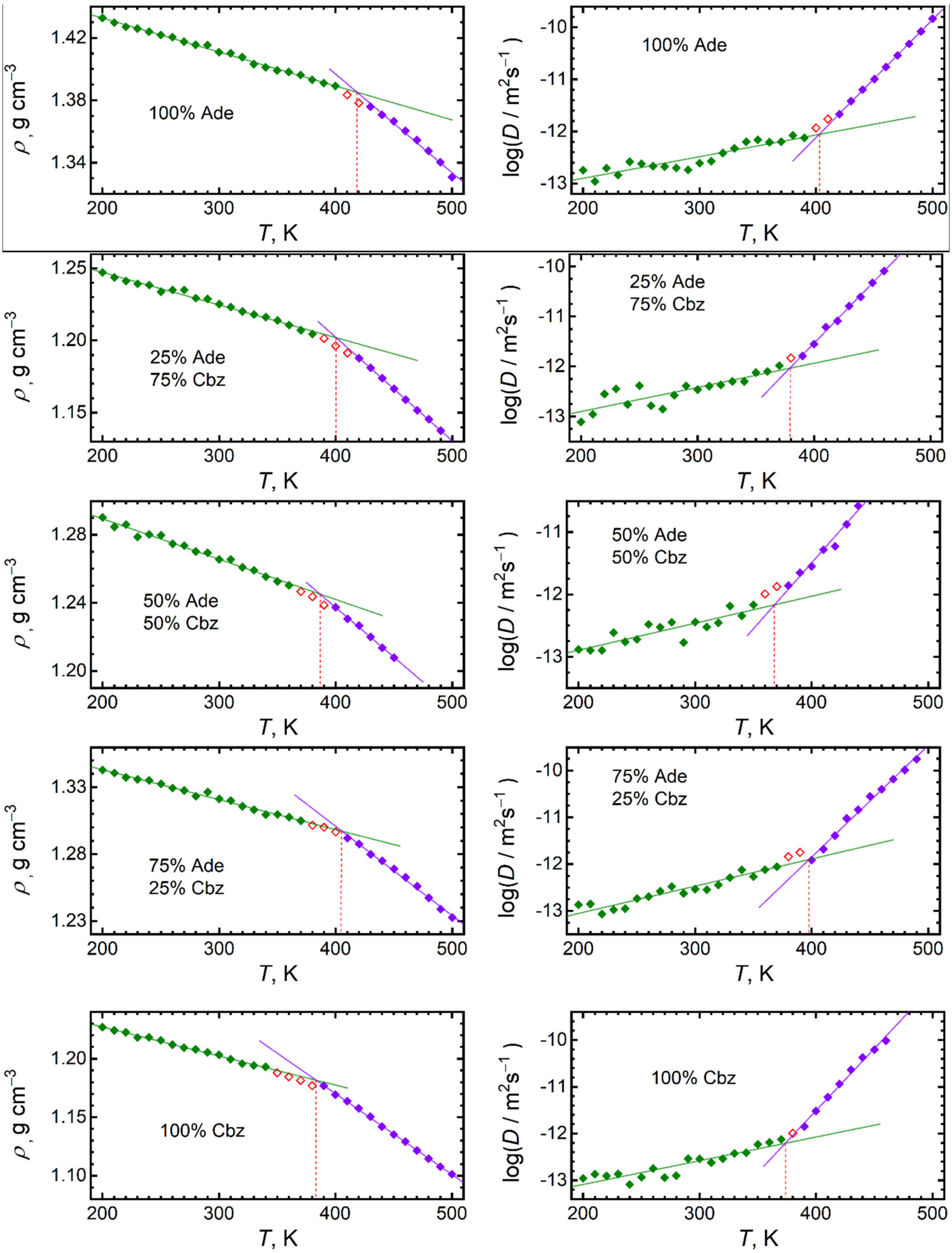

Figure S5. Determination of the glass transition temperatures (T_g) by the trend shift method for mixtures of adenine, Ade with carbamazepine, Cbz.

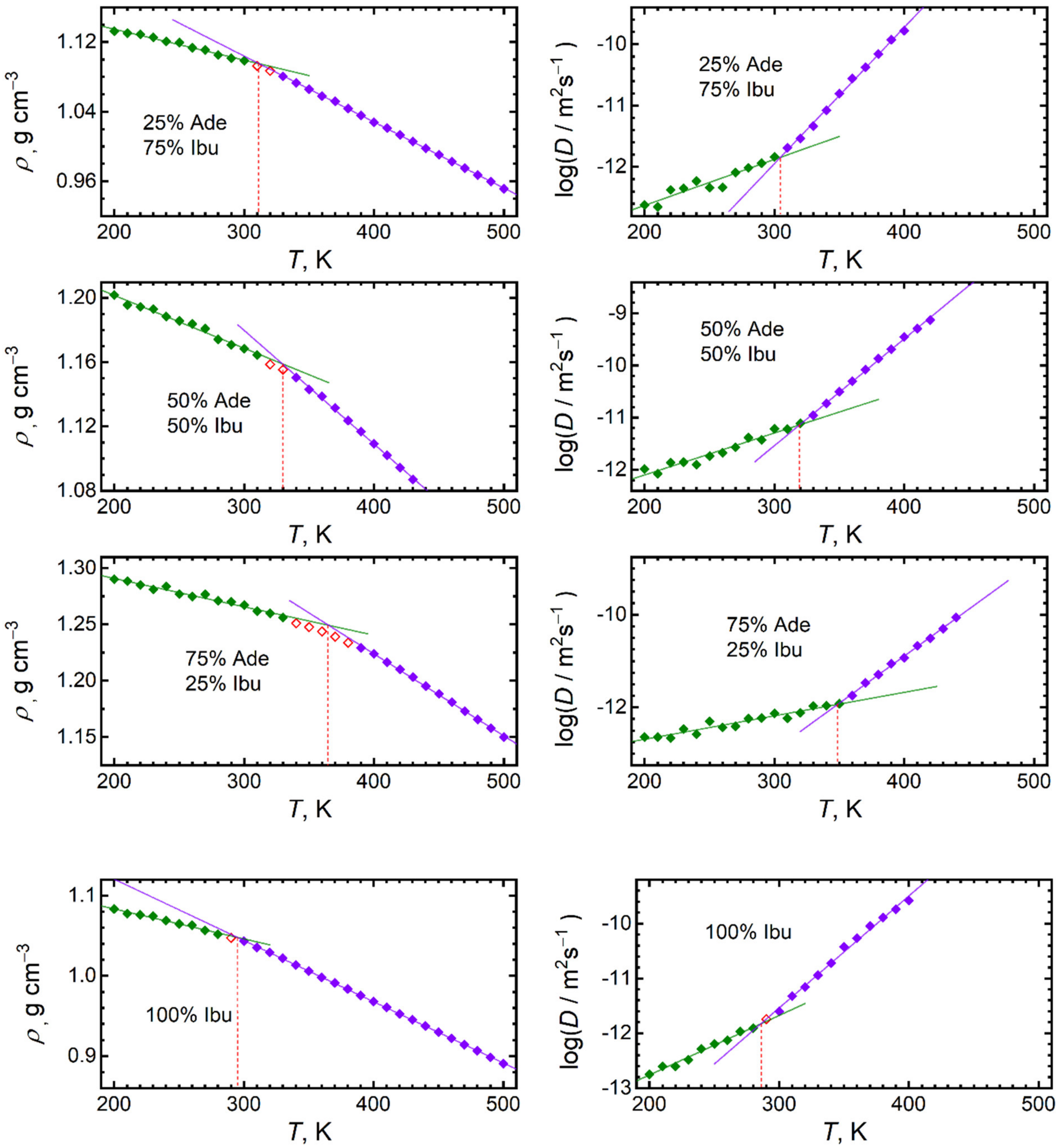

Figure S6. Determination of the glass transition temperatures (T_g) by the trend shift method for mixtures of adenine, Ade with RS-ibuprofen, Ibu.

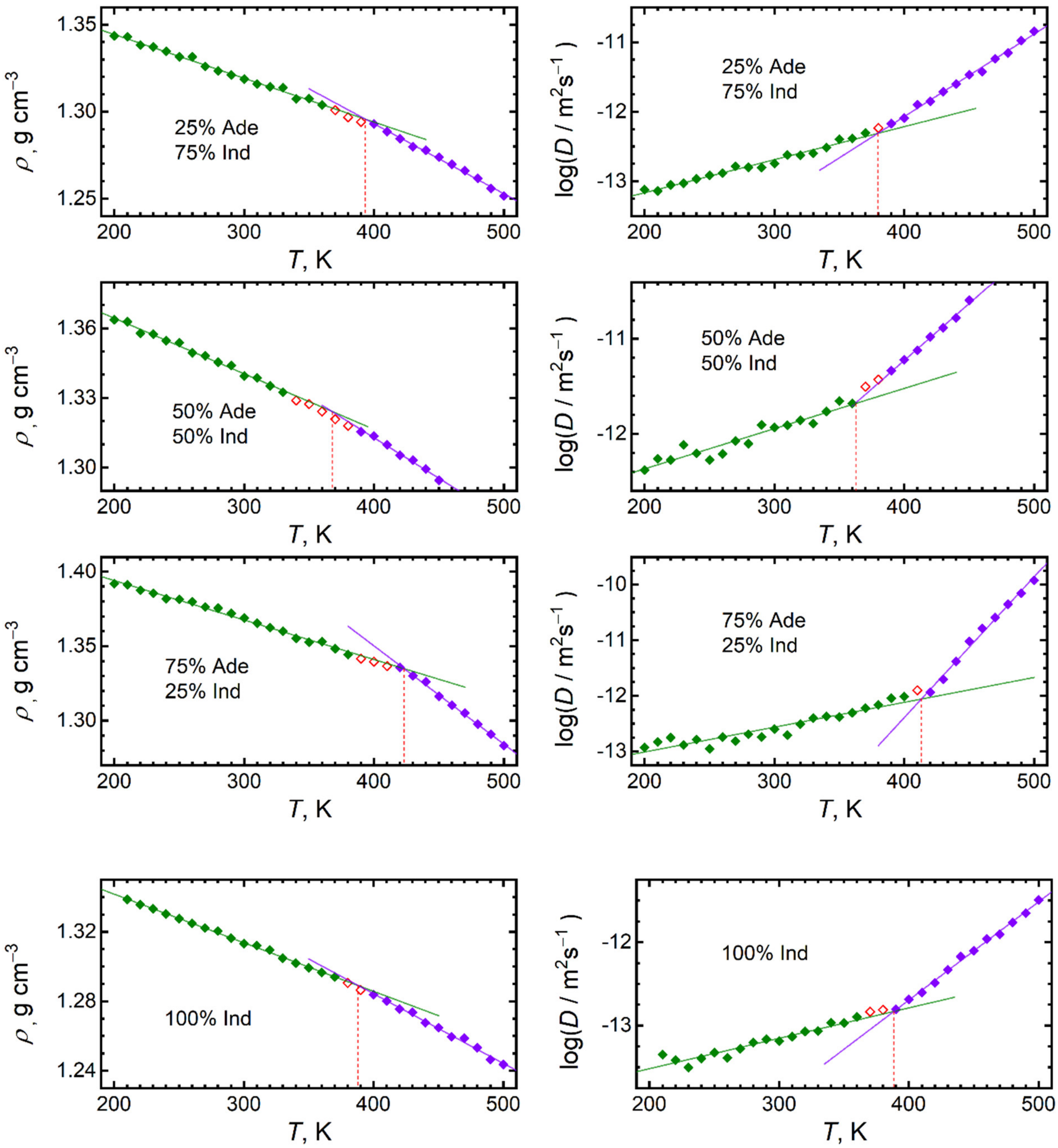

Figure S7. Determination of the glass transition temperatures (T_g) by the trend shift method for mixtures of adenine, Ade with indomethacin, Ind.

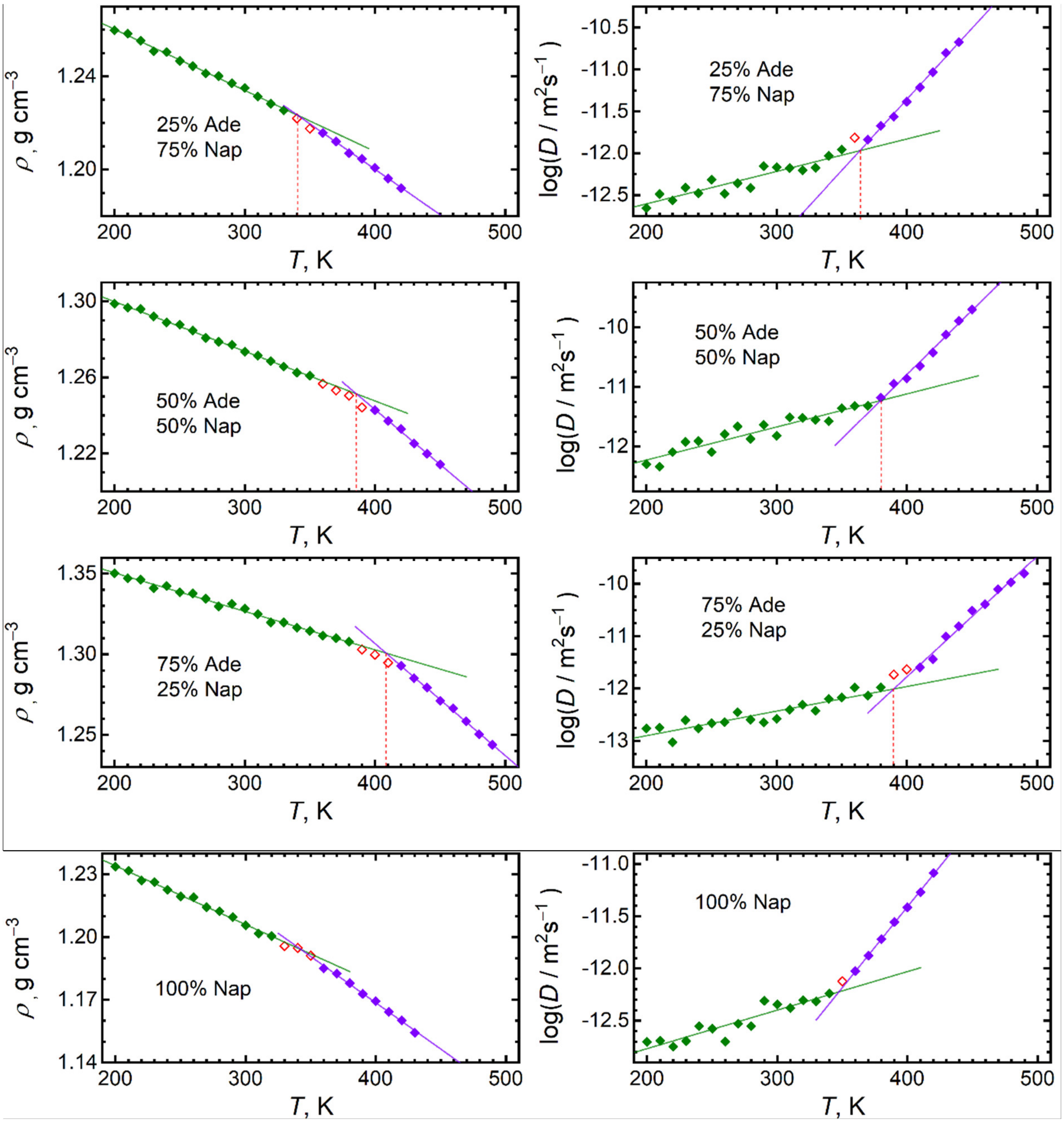

Figure S8. Determination of the glass transition temperatures (T_g) by the trend shift method for mixtures of adenine, Ade with naproxen, Nap.

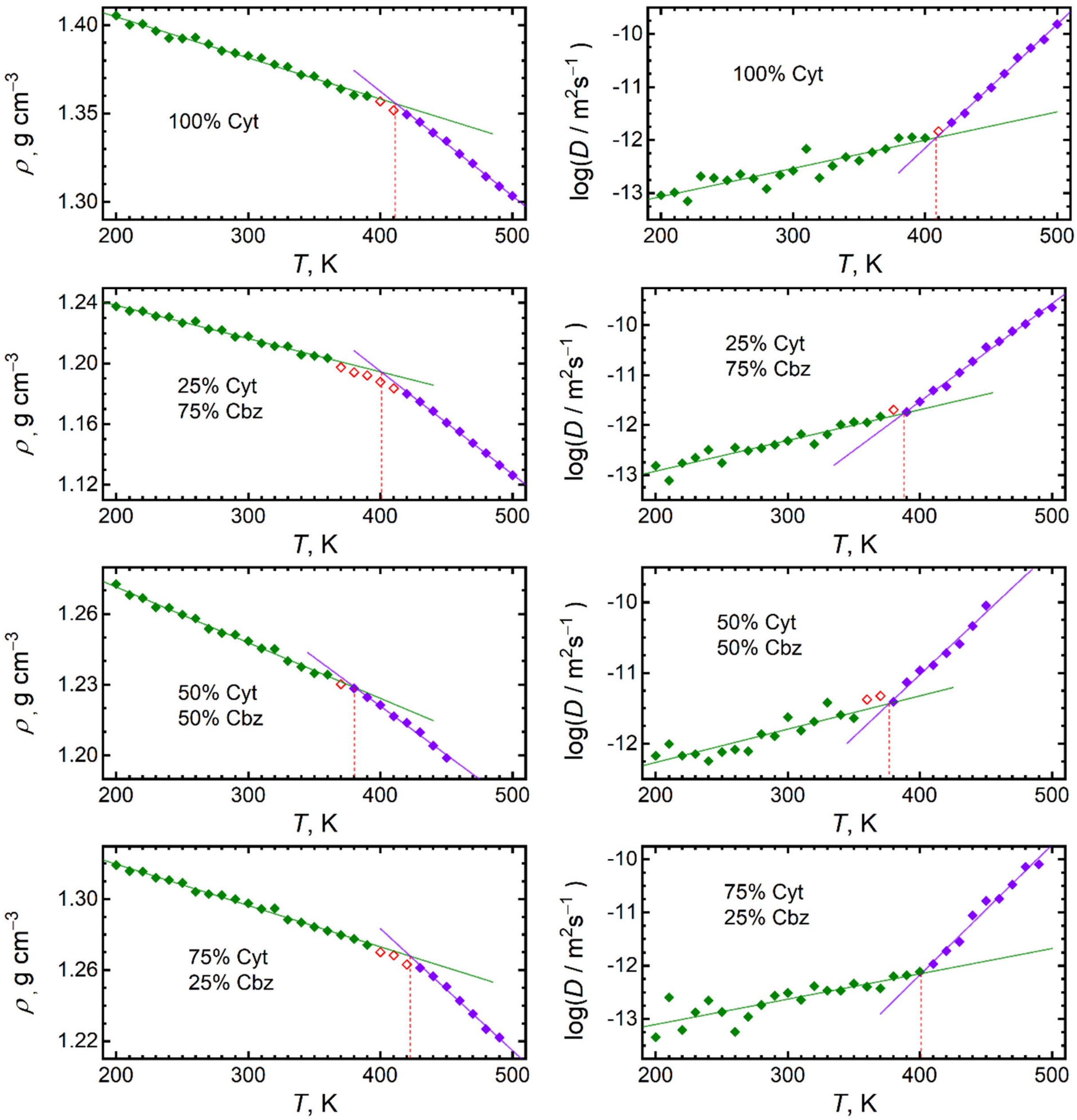

Figure S9. Determination of the glass transition temperatures (T_g) by the trend shift method for mixtures of cytosine, Cyt with carbamazepin, Cbz.

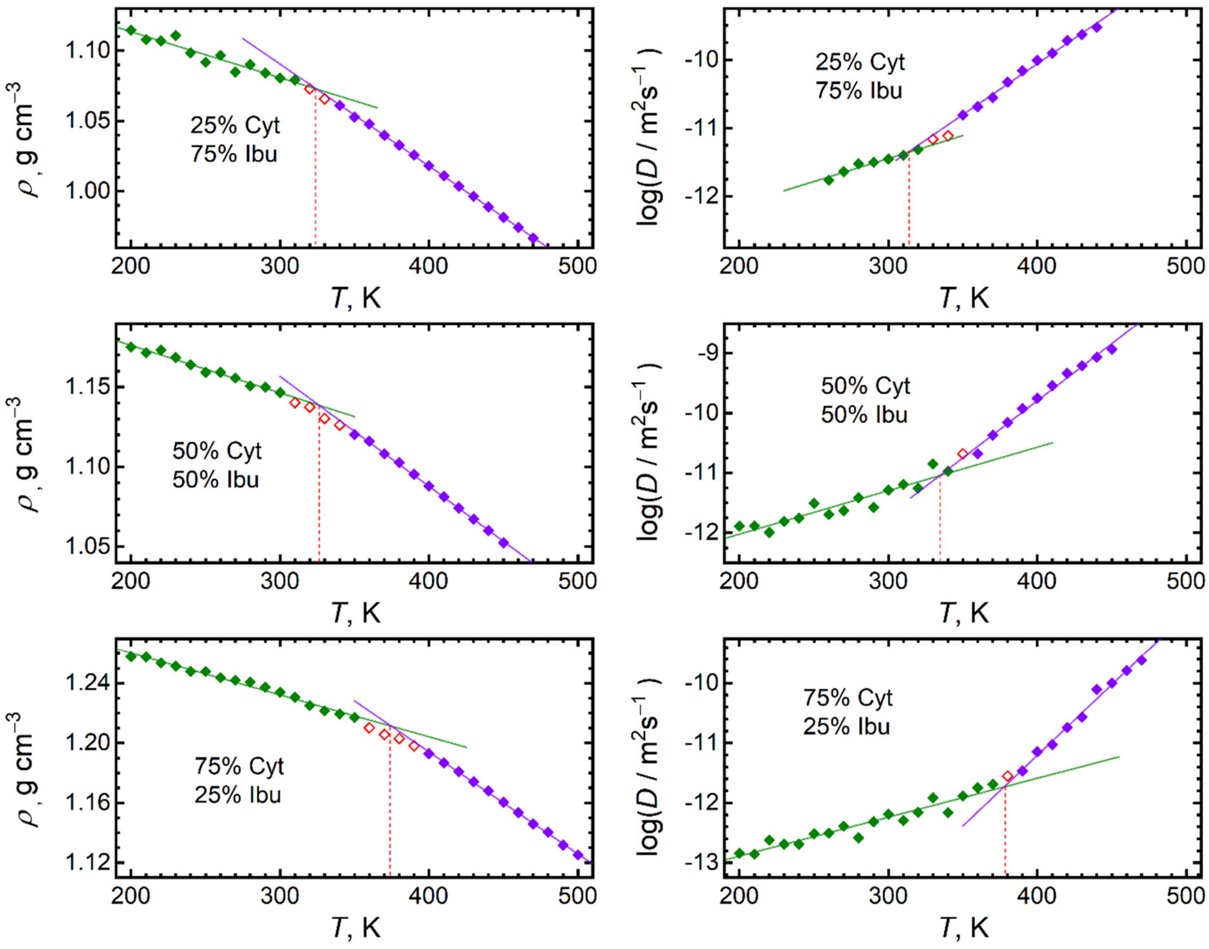

Figure S10. Determination of the glass transition temperatures (T_g) by the trend shift method for mixtures of cytosine, Cyt with RS-ibuprofen, Ibu.

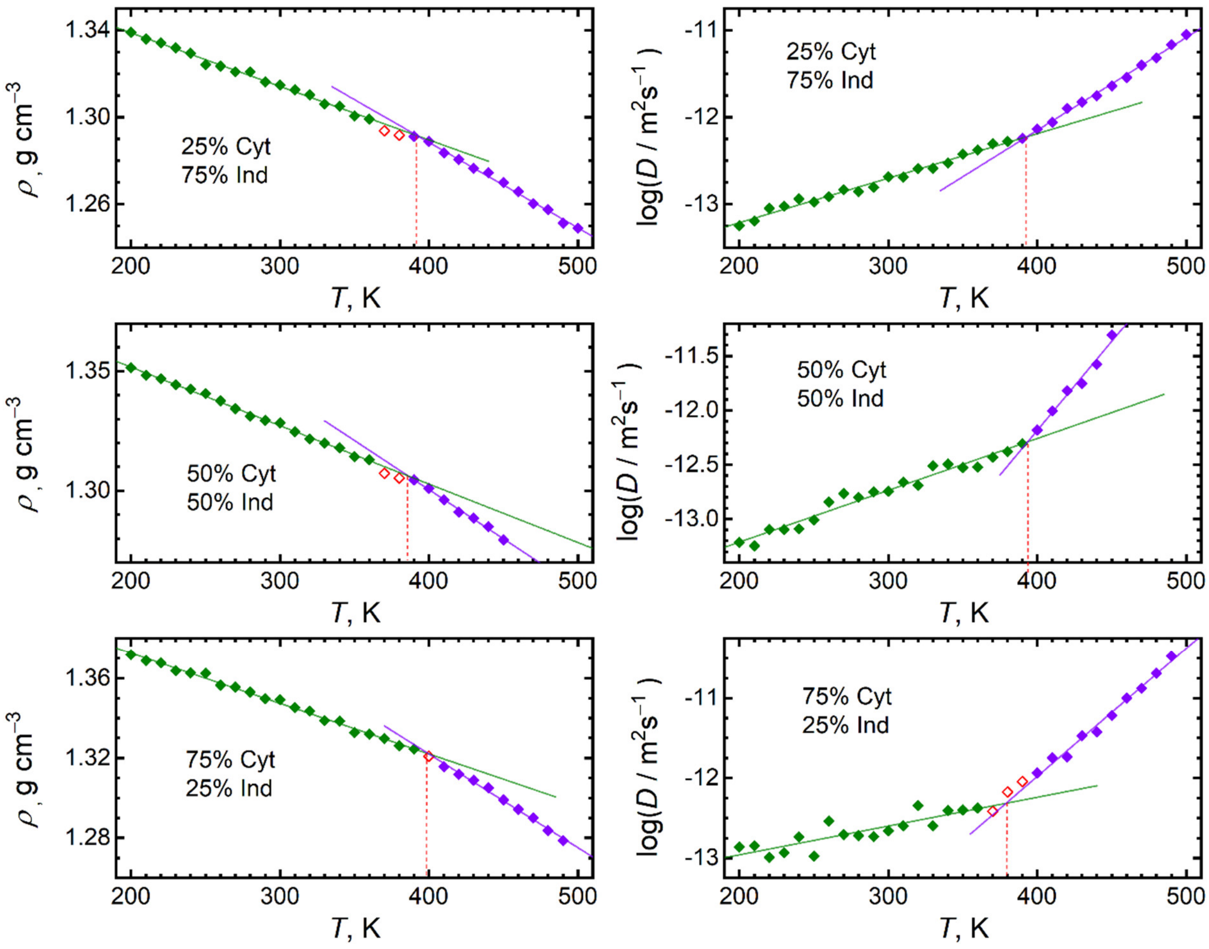

Figure S11. Determination of the glass transition temperatures (T_g) by the trend shift method for mixtures of cytosine, Cyt with indomethacin, Ind.

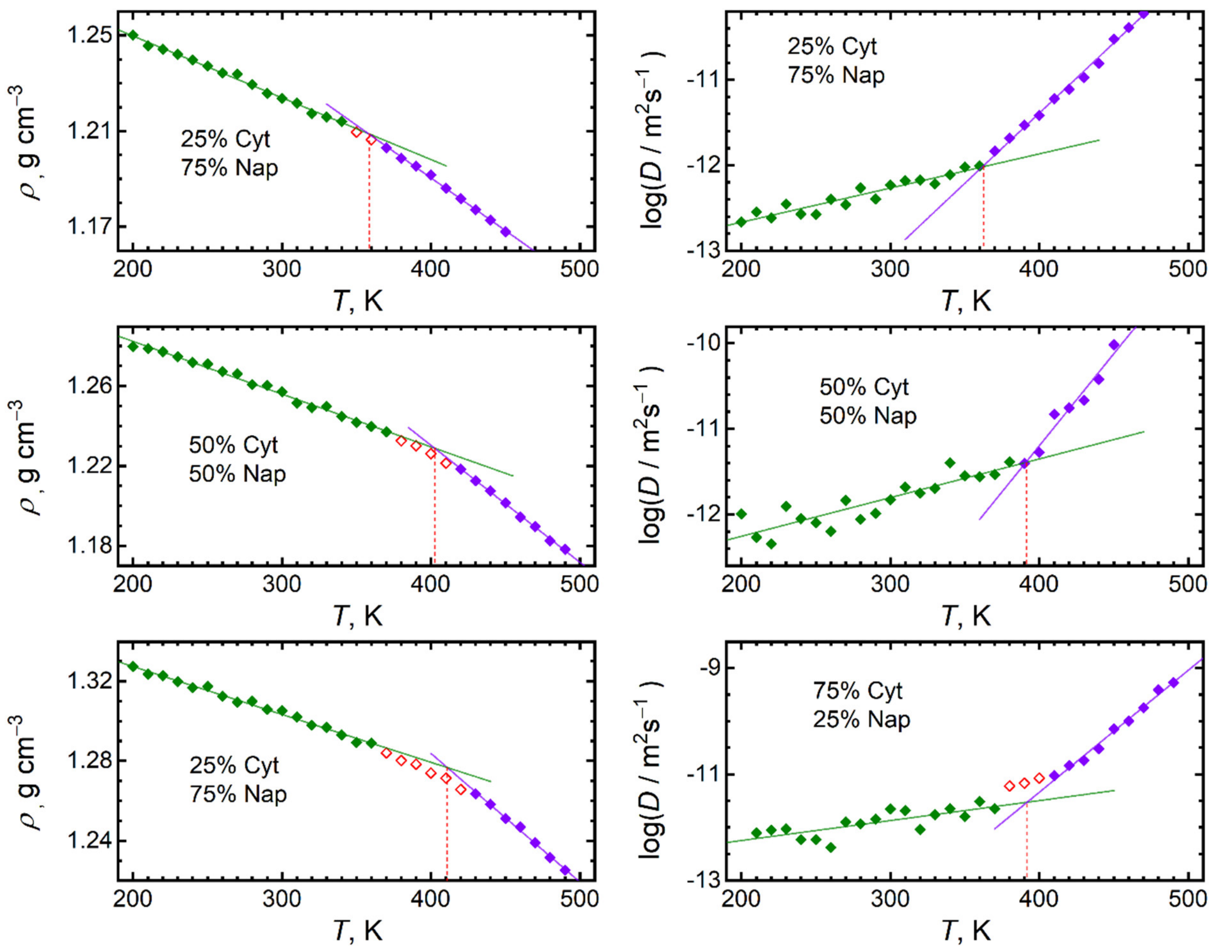

Figure S12. Determination of the glass transition temperatures (T_g) by the trend shift method for mixtures of cytosine, Cyt with naproxen, Nap.
